# Real-World Quality-of-Life Data in Metastatic Breast Cancer Patients Treated with CDK4/6 Inhibitors Using Four Assessment Tools

**DOI:** 10.3390/cancers17050818

**Published:** 2025-02-26

**Authors:** Ioana-Miruna Stanciu, Cornelia Nitipir

**Affiliations:** 1 Department of Oncology, “Carol Davila” University of Medicine and Pharmacy, 020021 Bucharest, Romania; 2 Oncology Department, Elias University Emergency Hospital, 011461 Bucharest, Romania; 3 Oncology Department, “Prof. Dr. Agrippa Ionescu” Emergency Clinical Hospital, 707961 Balotesti, Romania

**Keywords:** metastatic breast cancer, CDK4/6 inhibitors, quality of life, real-world data, questionnaires

## Abstract

Purpose: To evaluate the impact of the type of CDK4/6 inhibitor administered and comorbidities on the quality of life in patients with metastatic breast cancer, as well as the correlation between quality of life and patient outcomes. Materials and Methods: This prospective single-center study utilized four internationally validated questionnaires: the EORTC QLQ-C30 (European Organization for Research and Treatment of Cancer Core Cancer Quality of Life Questionnaire), the Depression, Anxiety, and Stress Scale-21 (DASS-21), the Multidimensional Fatigue Inventory (MFI), and the Pittsburgh Sleep Quality Index (PSQI), administered to a cohort of 76 patients undergoing treatment for metastatic breast cancer with CDK4/6 inhibitors. Results: Ribociclib is associated with fewer insomnia problems and appears to provide better sleep quality compared to other CDK4/6 inhibitors. Mental fatigue and loss of appetite negatively influence patient survival. Patients with comorbidities reported more severe insomnia and constipation. Comorbidities are associated with a lower quality of life, reflected in greater fatigue, insomnia, and constipation, as well as increased scores for depression and stress. Conclusions: Assessing global quality of life in metastatic breast cancer patients is essential, as it can predict patient progression and should be integrated into every breast cancer unit.

## Simple Summary

Patients with breast cancer endure psychological and physical symptoms that negatively impact their quality of life. This study underscores the importance of addressing the needs of metastatic breast cancer patients through four assessment tools that evaluate the patient from different perspectives: general quality of life, depression/anxiety/stress, fatigue, and sleep quality. To the best of our knowledge, this is the only study in Europe to utilize four distinct evaluation instruments to assess quality of life in a real-world context for patients with metastatic breast cancer undergoing treatment with CDK4/6 inhibitors. Our findings demonstrate that the quality of life for these patients is affected in various ways, particularly in their physical, social, and role functioning. Consequently, we encourage all medical oncologists to incorporate quality of life evaluation tools into their daily practice.

## 1. Introduction

Breast cancer is a heterogeneous disease, with treatment decisions and prognosis traditionally guided by immunohistochemistry (IHC) markers such as estrogen receptor (ER), progesterone receptor (PR), human epidermal growth factor receptor 2 (HER2), and Ki67 (a proliferation index marker), along with tumor size, tumor grade, and nodal status [1]. Of all patients with stage IV breast cancer, approximately 75% are hormone receptor-positive (HR+) and human epidermal growth factor receptor 2-negative (HER2−) [2].

The current treatment paradigm for HR+/HER2− advanced breast cancer involves sequencing endocrine therapy, targeted therapy, and/or chemotherapy to prolong patients’ lives, delay disease progression, and minimize cancer-related symptoms [3]. Cyclin-dependent kinase 4 and 6 inhibitors (CDK4/6 inhibitors) are rapidly transforming this treatment landscape. Currently, three CDK4/6 inhibitors have been approved by the US Food and Drug Administration: palbociclib, ribociclib, and abemaciclib [4,5,6].

Over the past decade, two significant developments in cancer medicine have emerged. The first is the understanding that cancer treatment depends on the patient’s overall health. The second is evaluating their well-being using psychosocial and quality of life (QOL) surveys. QOL has been an inferred medicinal outcome [7] since the time of Hippocrates. The first attempt by physicians to objectively evaluate how cancer treatments affect patients’ quality of life, rather than merely their quantity of life, was documented by Karnofsky et al. [8] in 1948. Clinical trials currently employ QOL tools to screen for psychological morbidity, predict survival, and measure treatment response [9].

One of the main clinical and research questions has been how breast cancer treatment affects a patient’s quality of life. Research on interventions for women with breast cancer has focused on psychosocial and emotional issues for the past 25 years. Findings by Ganz and Goodwin [10] indicated that as the number of survivors has increased, patients with breast cancer have been evaluated using various quality of life (QOL) tools to compare the effects of the disease and its treatments to those of healthy women and individuals with other chronic illnesses.

**Figure 1:**
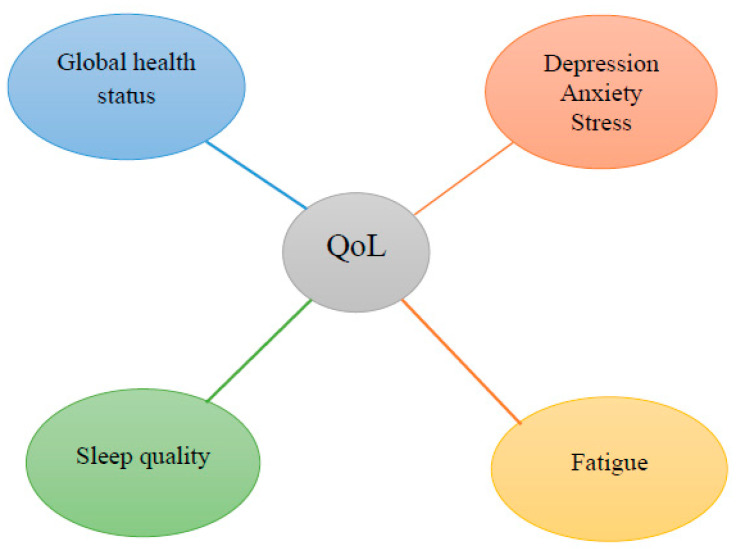
The four most important aspects of quality of life from our perspective.

However, as the diagnosis and treatment of the disease have improved significantly over time, quality of life has become an important outcome measure in breast cancer clinical investigations and survivorship studies [11]. To evaluate and summarize the existing evidence on quality of life in breast cancer patients, numerous questionnaires have been developed. Among these, we focused on the following validated instruments, which are among the most common and well-developed tools for measuring quality of life in breast cancer patients: the EORTC QLQ-C30 (European Organization for Research and Treatment of Cancer Core Cancer Quality of Life Questionnaire), the Depression, Anxiety, and Stress Scale-21 (DASS-21), the Multidimensional Fatigue Inventory (MFI), and the Pittsburgh Sleep Quality Index (PSQI). We chose to implement these questionnaires because we believe that the four most important aspects of quality of life (Figure 1)—global health status, emotional state, fatigue, and sleep quality—are very well comprised within these assessment tools.

Quality of life in patients with breast cancer is a crucial outcome. This paper presents an extensive overview of the topic ranging from descriptive findings in a specific population to real-world data regarding quality of life. Therefore, the aim of our study was to identify correlations between clinical and pathological characteristics and the quality of life in women with HR+/HER2− metastatic breast cancer. We outlined the patient’s real-world profile from clinical, biological, medical, and quality of life perspectives.

The four main quality of life surveys that we deemed pertinent to our study population are described in this original paper, along with the benefits, acceptability, and limitations. We have also discussed future directions and suggested solutions to address challenges related to questionnaire-type evaluations.

## 2. Materials and Methods

### Participants

**Figure 2:**
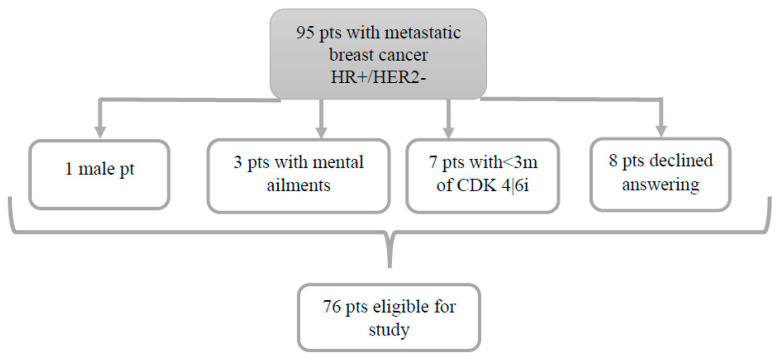
Flowchart diagram of the study.

This study was conducted on a group of 95 patients diagnosed with HR+/HER2− metastatic breast cancer and treated with CDK4/6 inhibitors. Out of these, 76 patients met the inclusion criteria, and the reported outcomes are derived from this carefully selected group (Figure 2). Three patients with psychiatric problems, seven patients with less than three months of therapy with CDK4/6i, eight patients who declined to answer, and one male patient were eliminated from the study.

Patient demographics and clinical characteristics were retrospectively extracted from electronic medical records during clinical evaluations at Elias University Emergency Hospital, Bucharest, Romania, from January 2018 to January 2024. Quality-of-life data were prospectively collected using the four questionnaires described below.

The study protocol was approved by the Ethics Committee of the Elias University Emergency Hospital, Bucharest, Romania. The study design, data analysis, interpretation, drafting, and revisions comply with the Helsinki Declaration and the Committee on Publication Ethics guidelines. All collected data were anonymized, considering the observational nature of the study, without personal data that could lead to the formal identification of the patient.

Inclusion criteria:

- Female patients aged ≥18 years.
- Either treated or currently in treatment with CDK4/6 inhibitors, with pathologically confirmed HR+/HER2− breast cancer.
- Minimum of three presentations in our oncology department.

We note that the respondents in our study are only patients who had received at least three months of therapy with one of the three CDK4/6 inhibitors (either palbociclib, ribociclib, or abemaciclib).

Exclusion criteria:

- Male patients or females aged < 18 years.
- Patients in critical condition or with mental/psychiatric ailments.
- Patients with < 3 months of CDK4/6 inhibitor therapy.
- Patients who did not complete all four questionnaires or declined answering all questions.

Data collected included age at diagnosis, area of residence, histological grade, stage at diagnosis (locally advanced vs. metastatic), metastasis localization, type of CDK4/6 inhibitor used, duration of CDK4/6 inhibitor therapy, associated endocrine therapy, menopausal status, Ki67 percentage, CA15-3 levels at diagnosis, comorbidities, survival status, family cancer history, and prior chemotherapy (before or after CDK4/6 inhibitor therapy).

## 3. Questionnaires

From January 2018 to January 2024, patients completed the four questionnaires via telephone after providing prior informed consent. Patients were given four questionnaires: the EORTC QLQ-C30 (European Organization for Research and Treatment of Cancer Core Cancer Quality of Life Questionnaire), the Depression, Anxiety and Stress Scale-21 (DASS-21), the Multidimensional Fatigue Inventory (MFI), and the Pittsburgh Sleep Quality Index (PSQI). Participation in the study was voluntary. Only patients with clear consciousness and no communication impairments were included. The patients were given the Romanian versions of the questionnaires. Both Romanian and English versions of the questionnaires are presented as Supplementary Materials. In this study, the Romanian version of the questionnaires was used, having been requested from each individual organization (EORTC group, Academic Medical Centre, University of Amsterdam Department of Medical Psychology Amsterdam, and Pittsburgh University). The DASS questionnaire is in the public domain, so permission is not needed for its use. All of these prestigious organizations confirm that the translations are conceptually equivalent to the original, culturally relevant to the context of the target country, and easily understood by the individuals to whom the translated instrument is administered.

### 3.1. EORTC QLQ-C30

The European Organization for Research and Treatment of Cancer Quality of Life Questionnaire (EORTC QLQ-C30) is a 30-item tool designed to assess various quality-of-life aspects in cancer patients. It was the first instrument developed by the EORTC QLG and is the product of over a decade of collaborative research. Following its general release in 1993, the QLQ-C30 has been utilized in several cancer clinical trials by a large number of research groups and in various non-trial studies [12]. Version 3.0 is currently the standard version of the QLQ-C30, and the one used in this study.

The QLQ-C30 comprises both multi-item scales and single-item measures covering the following:

- Five functional scales.
- Three symptom scales.
- A global health status/quality-of-life scale.
- Six single items.

Each multi-item scale includes a different set of items, with no item occurring in more than one scale. All scales and single-item measures range in score from 0 to 100. Scores range from 0 to 100, with higher functional and global health scores indicating better quality of life, while higher symptom scores denote greater symptom severity [13].

### 3.2. Depression, Anxiety, and Stress Scale-21 Items (DASS-21)

The DASS-21 is a set of three self-report scales designed to measure the emotional states of depression, anxiety, and stress.

Each of the three DASS-21 scales contains seven items, divided into subscales with similar content.

- Depression: Assesses dysphoria, hopelessness, devaluation of life, self-deprecation, lack of interest/involvement, anhedonia, and inertia.
- Anxiety: Measures autonomic arousal, skeletal muscle effects, situational anxiety, and subjective experiences of anxious affect.
- Stress: Evaluates chronic nonspecific arousal, difficulty relaxing, nervous arousal, irritability, and impatience.

Scores for depression, anxiety, and stress are calculated by summing the scores for the relevant items.

The DASS-21 is based on a dimensional rather than a categorical concept of psychological disorder. The assumption underlying the DASS-21 development (which has been confirmed by research data) is that the differences between depression, anxiety, and stress experienced by normal subjects and clinical populations are essentially differences in degrees [14].

The questionnaire’s 21 items consist of three self-reported measures intended to evaluate DASS. The four possible scores for the seven items on the scales are as follows:

- “Did not apply to me at all”.
- “Applied to me to some degree or some of the time”.
- “Applied to me to a considerable degree or a good part of the time”.
- “Applied to me very much or most of the time”.

Each item is rated on a Likert scale from 0 to 3. Scores of related items are summed to determine the depression, anxiety, and stress scores. According to the manual, the resulting ratings are classified as: “normal, mild, moderate, severe, or extremely severe” [14].

### 3.3. Multidimensional Fatigue Inventory (MFI)

The MFI is a 20-item scale designed to evaluate five dimensions of fatigue: general fatigue, physical fatigue, reduced motivation, reduced activity, and mental fatigue. It has been utilized in various participant populations, including cancer patients, with an average age of 61 years.

Respondents use a scale ranging from 1 to 5 to indicate how well certain statements regarding fatigue represent their experiences. Several positively phrased items are reverse-scored. Higher total scores correspond to more acute levels of fatigue [15].

The questionnaire has 20 questions rated on a 5-point scale (1 = “yes, that is true” to 5 = “no, that is not true”).

Items are scored 1–5, with ten positively phrased items reverse-scored (items 2, 5, 9, 10, 13, 14, 16, 17, 18, and 19). In the final score, high scores represent more fatigue. The final score is divided into the following subscales:

- General fatigue: Items 1, 5, 12, and 16.
- Physical fatigue: Items 2, 8, 14, and 20.
- Reduced activity: Items 3, 6, 10, and 17.
- Reduced motivation: Items 4, 9, 15, and 18.
- Mental fatigue: Items 7, 11, 13, and 19.

### 3.4. Pittsburgh Sleep Quality Index (PSQI)

The PSQI is a 19-item self-reported questionnaire designed to assess sleep quality over the past month. The items are grouped into seven components:

1. Subjective sleep quality.
2. Sleep latency.
3. Sleep duration.
4. Habitual sleep efficiency.
5. Sleep disturbances.
6. Use of sleeping medication.
7. Daytime dysfunction.

Five additional questions rated by the respondent’s roommate or bed partner are included for clinical purposes and are not scored. The questionnaire has been validated with various clinical populations, including patients with major depressive disorder, disorders of initiating and maintaining sleep, disorders of excessive somnolence, cancer, and fibromyalgia [16].

Each component score of the PSQI ranges from 0 to 3, with 3 indicating the greatest dysfunction or disturbance. The seven component scores are summed to obtain a global PSQI score, which ranges from 0 to 21. Higher scores indicate poorer sleep quality, with a score greater than 5 suggesting significant sleep difficulties.

## 4. Statistical Analysis

All questionnaire data were collected on paper and entered into Microsoft Excel by an independent researcher. Statistical processing was conducted using IBM SPSS Statistics for Windows, Version 29.0 (30-day trial version, Armonk, NY, USA: IBM Corp.).

- Nominal data: Presented as absolute frequency and percentage.
- Continuous variables: Expressed as mean, standard deviation, minimum, and maximum.

Analysis of the association between categorical variables was conducted using cross-tabulation and the χ^2^ (chi-square) test. If the results of the chi-square test were altered enough to be considered, Fisher’s exact test was used. The Mann–Whitney U test was used to compare means for dichotomous variables. The Kruskal–Wallis H test compared means between groups, as the variables had a non-Gaussian distribution. A *p*-value of < 0.05 was considered statistically significant.

In this study, we used bootstrapped 95% confidence intervals (CIs) to assess the variability and reliability of clinical outcome measures among oncology patients with and without comorbidities. The bootstrap method was employed with 1000 resamples, allowing us to generate robust estimates of the mean and confidence intervals without assuming normality. The interquartile range (IQR) was used as a measure of dispersion for skewed distributions, in line with best practices in biostatistics. A horizontal bar plot with confidence intervals was constructed to visually compare the two groups, with blue representing patients without comorbidities and red representing those with comorbidities. The mean values were plotted as discrete points, while the 95% CIs were illustrated as horizontal lines, providing a clear depiction of the uncertainty around the estimates. The overlap or separation of confidence intervals was used to qualitatively assess the presence of statistically meaningful differences between the groups.

To account for the potential inflation of Type I error due to multiple comparisons, we applied false discovery rate (FDR) correction using the Benjamini–Hochberg procedure. This method controls the expected proportion of false positives while maintaining statistical power. Adjusted *p*-values were calculated for all analyses involving multiple testing, ensuring a more rigorous interpretation of statistical significance. *p*-values below 0.05 after FDR correction were considered statistically significant.

## 5. Results

Patients’ demographics and clinical characteristics are shown in Table 1.

The most frequent comorbidities that we encountered were as follows:

- Hypertension (30 patients).
- Type II diabetes (16 patients).
- Cardiovascular comorbidities (16 patients).
- Dyslipidemia (15 patients).
- Obesity (13 patients).
- Osteoporosis (9 patients).
- Hypothyroidism (6 patients).
- Depressive syndrome (6 patients).
- Chronic kidney disease (5 patients).
- Hepatic steatosis (4 patients).

Other comorbidities that we encountered in the case of patients in this study were the following: cataract, tuberculosis in history, respiratory failure, Parkinson’s disease, hiatal hernia, polynodular goiter, chronic obstructive pulmonary disease, pulmonary fibrosis, dementia, fractures on pathological bone, polyneuropathy, autoimmune thyroiditis, stroke, hearing loss, right calf amputation, diabetic nephropathy, gallbladder stones. These comorbidities were encountered with a frequency of one or two cases in our study population.

We need to mention that breast cancer was the cause of all deaths, despite the fact that these patients also had many major comorbidities.

The analysis of quality of life, as derived from the four questionnaires, was correlated with the type of CDK4/6 inhibitor received, patient’s evolution (death vs. survival), and associated comorbidities. Three multivariate analyses were conducted to identify statistically significant differences among patient groups. The findings of each analysis are evaluated separately in the following subchapters.

### 5.1. Overall Analysis of Quality of Life (QoL)

**Figure 3:**
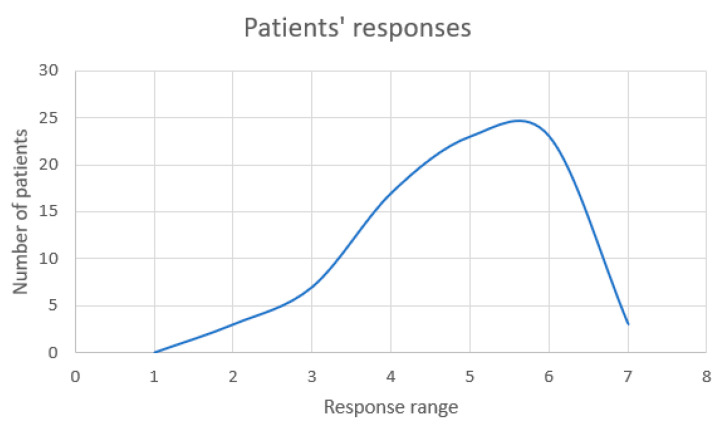
Analysis of patients’ responses to the question “How would you rate your overall health during the past week?”.

**Figure 4:**
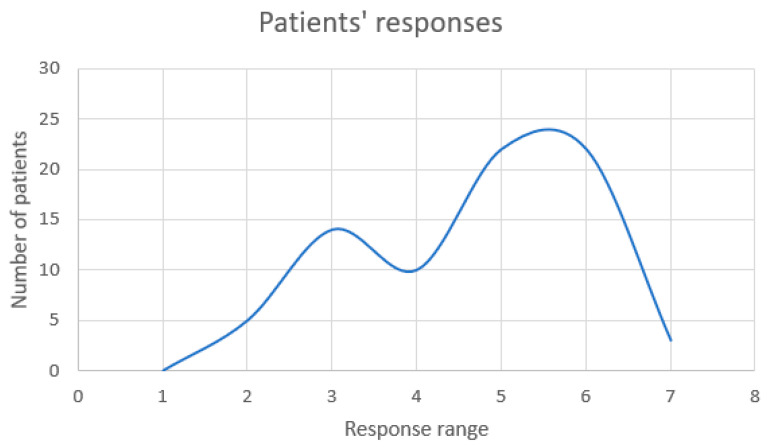
Analysis of patients’ responses to the question “How would you rate your overall quality of life during the past week?”.

The following statistics (Table 2) summarize overall health status, functioning, physical and emotional symptoms, sleep quality, and stress/anxiety levels. For improved accuracy, data from the four questionnaires were consolidated into the SPSS database, allowing for comprehensive statistical analysis. The findings were divided into subgroups as follows:

The final two single items on the EORTC-QLC-C30 tool assessment are “How would you rate your overall health during the past week?” and “How would you rate your overall quality of life during the past week?”, ranging from 1 (poor) to 7 (excellent) in response. These two questions rank among the most important when evaluating quality of life, and the answers from our patients are displayed in the following figures (Figure 3 and Figure 4).

With an answer of 5 (*n* = 23) or 6 (*n* = 23) out of 7, which is the highest health quality, the majority of patients (*n* = 46) believed that their general health had been good over the last several weeks.

In terms of subjective perception and quality of life, the majority of our patients (*n* = 44) believed they had a good quality of life, with responses ranging from 5 (*n* = 22) to 6 (*n* = 22) out of 7, which is regarded as an exceptional quality of life.

**Figure 5:**
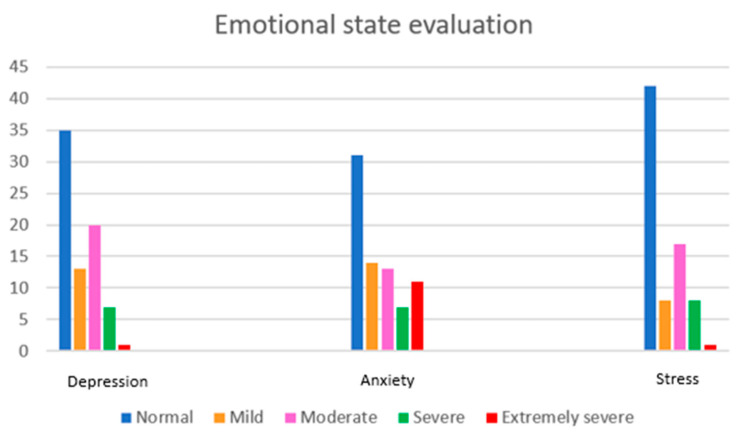
Analysis of the three emotional states: depression, anxiety, and stress.

The three emotional states—stress, anxiety, and depression—are also graphically analyzed (Figure 5). There are patients who may have mild, moderate, severe, or extremely severe emotions, with a wide range and variability across patients. However, the majority of respondents appear to have no issues related to depression (*n* = 35), anxiety (*n* = 31), or stress (*n* = 42).

### 5.2. Quality of Life Analysis Correlated with CDK4/6 Inhibitor Type

The following analysis (Table 3; Table S1: the Kruskal–Wallis H test statistics which compares means between groups for the quality of life analysis correlated with CDK4/6i test statistics can be found in the Supplementary Material) examines how each item of the four questionnaires correlates with the type of CDK4/6 inhibitor (palbociclib, ribociclib, and abemaciclib).

Differences between groups regarding insomnia, diarrhea, and sleep quality were the only significant ones. All other correlations and differences between groups were not statistically significant.

### 5.3. Analysis of Quality of Life Correlated with Patient Evolution

The following statistics (Table 4; Table S2: the Kruskal–Wallis H test statistics which compares means between groups for the quality of life analysis correlated with patient evolution test statistics can be found in the Supplementary Material) analyze how each item of the four questionnaires correlates with patient evolution (survival vs. death):

Below is a summary table (Table 5) showing the point-biserial correlation coefficient (r_p_b) for each variable comparing survival (coded as 1) versus death (coded as 0). For each variable, the coefficient was computed using the following formula:

r_p_b = (M_1_ − M_0_)⁄s_p_ × √(p · q)

where M_1_ and M_0_ are the group “means” (using the provided medians as surrogates), s_p_ is the pooled standard deviation, and p and q are the proportions in the survival and death groups, respectively.

**Figure 6:**
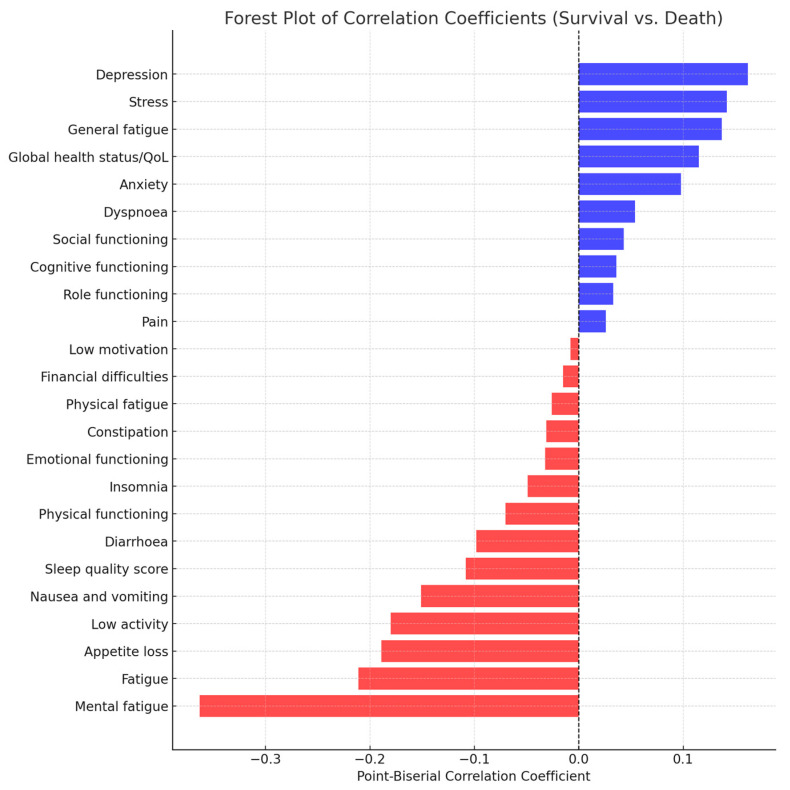
Forest plot of correlation coefficients for survival vs. death. Positive correlations (blue bars) indicate that higher scores on that variable are associated with survival. Negative correlations (red bars) indicate that higher scores on that variable are associated with death. The dashed vertical line at zero represents no association.

The next figure (Figure 6) is the graphical representation of the correlation coefficients for survival vs. death.

### 5.4. Analysis of Quality of Life Correlated with Comorbidities

**Figure 7:**
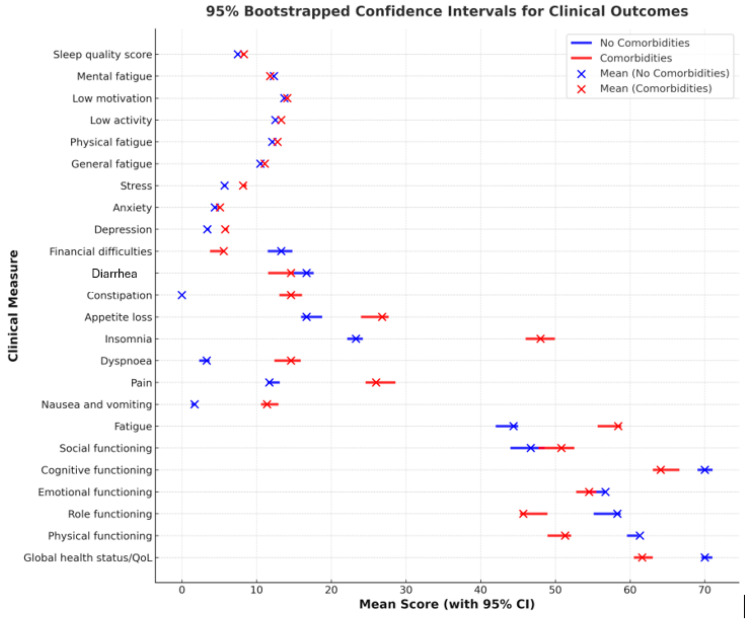
Bootstrapped 95% confidence intervals for clinical outcomes in oncology patients with and without comorbidities. Blue (No Comorbidities): represents the mean scores and 95% confidence intervals (CIs) for patients without comorbidities. Red (Comorbidities): represents the mean scores and 95% confidence intervals (CIs) for patients with comorbidities. Each horizontal line represents the range of the 95% confidence interval (CI) for a given clinical measure, while the dots indicate the mean values.

The following statistics (Table 6; Table S3: the Kruskal–Wallis H test statistics which compares means between groups for the quality of life analysis correlated with comorbidities test statistics can be found in the Supplementary Material) analyze how each item of the four questionnaires correlates with the comorbidities present in the patients:

In the figure below (Figure 7), we analyzed bootstrapped 95% confidence intervals for clinical outcomes in oncology patients with and without comorbidities.

### 5.5. Analysis of Quality of Life During COVID Pandemic

We acknowledge that the pandemic introduced unique challenges in oncology care, including delays in diagnosis and treatment, modifications in therapeutic approaches, and disruptions in healthcare access, all of which could influence progression-free survival (PFS), overall survival (OS), and time on treatment (ToT).

To address this concern, we conducted an analysis specifically examining the impact of COVID-19 infection on PFS, OS, and ToT within our cohort. The findings from this analysis have been incorporated into Table 7 and Table 8, allowing for a more comprehensive understanding of the potential confounding effects introduced by the pandemic.

We believe that taking the COVID pandemic period into consideration strengthens the robustness of our study by considering external factors that may have influenced treatment outcomes.

Time on treatment (ToT) is defined as the duration a patient remains on CDK4/6i therapy before discontinuation, regardless of the reason for stopping treatment. Progression-free survival (PFS) is defined as time from treatment initiation to disease progression or death. Overall survival (OS) is defined as time from diagnosis or treatment initiation to death from any cause.

Patients with COVID infection had a significantly longer duration of treatment (26 months) than those without infection (13.44 months; *p* = 0.002). Regarding PFS and OS, no significant differences were identified (Table 7 and Table 8).

## 6. Discussions

Quality of life is affected in multiple dimensions, particularly in physical, social, and role functioning. Fatigue is a central symptom that impacts physical functioning and motivation. Sleep problems are common and correlated with other symptoms, such as fatigue and stress. Financial difficulties and nausea are less common compared to other issues. Levels of stress and depression range from moderate to high, with significant variations between patients.

Differences between groups regarding insomnia and diarrhea are significant. Ribociclib is associated with fewer insomnia problems, while abemaciclib is linked to more frequent diarrhea. Ribociclib appears to provide better sleep quality compared to other treatments.

Indicators with statistically significant differences regarding patient evolution (death vs. survival) include loss of appetite (*p* = 0.036)—those who died had higher scores—and mental fatigue (*p* = 0.003)—those who died had higher scores. Indicators with nearly significant differences include fatigue (*p* = 0.094), with higher scores in those who died, and nausea and vomiting (*p* = 0.088), with higher scores in those who died. These results suggest that mental fatigue and loss of appetite may have a greater influence on patient survival and warrant further investigation in the context of symptom management.

Indicators with statistically significant differences in the analysis of comorbidities include insomnia (*p* = 0.014)—people with comorbidities reported more severe insomnia—and constipation (*p* = 0.046). Nearly significant indicators include fatigue (*p* = 0.091), with higher scores in those with comorbidities, depression (*p* = 0.075), and stress (*p* = 0.121).

Comorbidities are associated with a lower quality of life, reflected in greater fatigue, insomnia, and constipation, as well as increased scores for depression and stress. These differences indicate the need for special attention to symptom management and psychological health in patients with comorbidities.

### 6.1. Patient Profile from a Clinical, Biological, Treatment, and Quality-of-Life Perspective

Quality of life was moderate-to-good (62.72 ± 19.93), but physical (52.63 ± 25.96) and social (50.22 ± 37.96) functioning were significantly affected. Emotionally, patients were at a moderate level (54.82 ± 23.50), with large variations between cases. The main symptoms included fatigue (56.58 ± 26.37) and insomnia (44.74 ± 30.58), which had a significant negative impact. Pain (24.12 ± 29.61) and loss of appetite (25.44 ± 29.75) were present but moderate on average. Cognitive functioning (64.91 ± 27.83) was better preserved than in other domains, but the social score indicated isolation for some patients. Effective symptom management (e.g., fatigue, insomnia) and social support are essential for improving quality of life.

### 6.2. Benefits of Quality of Life Assessment in Breast Cancer Patients

One significant advantage of quality of life assessment, according to Greenhalgh and Meadows, Di Maio and Perrone, and Le et al., is that it promotes shared decision-making and facilitates communication between doctors and patients [17,18,19,20] by providing patients with feedback on their expectations, goals, and progress [21].

Additional potential benefits of quality-of-life testing include establishing a baseline evaluation when initiating treatment or therapy, identifying treatable problems that may otherwise be overlooked in routine patient care, and addressing crucial supportive measures such as illness education and dietary counseling. These aspects can play a vital role in enhancing patient well-being, treatment adherence, and overall quality of care. Measuring quality of life may also be useful in detecting additional mental and physical issues and tracking declines in functional ability.

### 6.3. Acceptability of Quality of Life Assessments

Quality of life assessments are deemed acceptable by both patients and healthcare providers for assisting individuals with breast cancer in addressing aspects of their quality of life that have been impacted by the disease. Numerous studies have illustrated the rationale behind doctors’ acceptance of the use of quality of life measures.

According to Stiggelbout et al. [22], patients’ emotions about quality of life, as opposed to quantity of life, can be assessed using questionnaires. In their study on patient acceptability of quality of life questionnaires, Apolone et al. [23] found that 64% of patients responded to the surveys, with very few items missing. These findings demonstrate that quality of life questionnaires, specifically the EORTC QLQ-C30 used in this study, are acceptable to patients [23].

### 6.4. Limitations of Quality of Life Assessments

Assessments with fewer answer alternatives and verbal questions, rather than solely numerical ones, were deemed acceptable by cancer patients. Furthermore, research by Pijls-Johannesma et al. has shown that respondents preferred questionnaires that did not include excessively sensitive, personal, or irrelevant questions [24].

This could be one of the limitations of our study, given that the majority of answers were numerical, some questions were too personal (e.g., those regarding sexual life), and some questions were irrelevant to specific subgroups of patients. For instance, since many respondents were retired, inquiries about work-related issues were irrelevant.

Another major limitation is the reluctance of doctors to use the tests due to logistical issues, such as time and budget constraints [25,26]. Moreover, physicians are often less familiar with quality of life assessments than with imaging or physiological tests and may be unsure how to interpret the results or respond to them [26].

Particularly in the elderly, compliance with questionnaires can be problematic due to cognitive disorders, comorbidities, and illiteracy. This is why one of the exclusion criteria in our study was patients in critical condition or those with mental or psychiatric ailments.

Another significant limitation of this study is that quality of life (QoL) was assessed at a single time point rather than longitudinally. As a result, we were unable to evaluate changes in QoL over time, such as before and after initiating systemic therapy with CDK4/6 inhibitors. A longitudinal assessment would provide a more comprehensive understanding of the impact of treatment on patient-reported outcomes.

Furthermore, several respondents felt embarrassed to answer certain questions in the presence of researchers. We assured them that their responses would remain confidential.

In conclusion, our review has several limitations that must be considered with caution when interpreting the results.

### 6.5. Future Directions and Suggested Solutions to Address Challenges in Questionnaire-Type Evaluations

Simply adding quality of life metrics to the everyday duties of oncology staff will not suffice for future implementation. Quality of life should be integrated into the care process of patient-reported outcomes by rethinking care delivery, particularly by considering the principles of effective distribution and establishing new infrastructures and technologies.

The ideal evaluation system must be clinically relevant (helpful and delivered on time), adaptable, culturally sensitive, low-burden, low-cost, integrated into standard operating processes, and meet community, consumer, and regulatory needs [27].

Patients, doctors, and office workers may be reluctant to adopt new practices due to time constraints. Rather than merely providing the doctor with a functional status score, offering interpretations and suggestions regarding the score and available resources could be more beneficial [28].

Overall, we believe breast cancer patients stand to benefit significantly from the use of quality of life evaluations in clinical practice. Physicians will be better equipped to make treatment decisions if they receive reliable information about health-related quality of life from self-reported questionnaires. Quality of life has become a more significant consideration in the holistic treatment of patients with breast cancer.

We firmly believe that all breast cancer units should implement these questionnaires, along with specialized personnel to administer them, interpret the results, and communicate findings to the medical staff.

## 7. Conclusions

To the best of our knowledge, this is the only study in Romania and throughout Europe to assess quality of life in a real-world setting for patients with metastatic breast cancer receiving CDK4/6 inhibitors using four different evaluation tools.

This study demonstrates that the EORTC QLQ-C30 (European Organization for Research and Treatment of Cancer Core Cancer Quality of Life Questionnaire), the Depression, Anxiety and Stress Scale-21 (DASS-21), the Multidimensional Fatigue Inventory (MFI), and the Pittsburgh Sleep Quality Index (PSQI) are practical and valid instruments for measuring quality of life in patients with advanced breast cancer. We encourage every medical oncologist to implement these questionnaires in their day-to-day practice.

## Figures and Tables

**Table 1:** The patients’ demographics and clinical characteristics.

Characteristics	*n* (%)
Age—median (±SD)	57.08 (±13.03)
Histological grade	
G1	8 (8.4%)
G2	63 (66.3%)
G3	18 (18.9%)
Non-metastatic/metastatic at diagnoisis	
Non-metastatic	56 (58.9%)
Metastatic	39 (41.1%)
Metastases	
M0	6 (6.3%)
M1 visceral	17 (17.9%)
M1OSS	26 (27.4%)
M1 visceral + M1OSS	43 (45.3%)
Others	3 (3.2%)
Treatment prior to CDK4/6i therapy	
no treatment	38 (40.0%)
chemotherapy	48 (50.5%)
hormonotherapy	6 (6.3%)
Treatment after CDK4/6i therapy	
no treatment	71 (74.7%)
chemotherapy	16 (16.8%)
hormonotherapy and chemotherapy	6 (6.3%)
The type of CDK4/6i administered	
Ribociclib	24 (25.3%)
Palbociclib	41 (43.2%)
Abemaciclib	30 (31.6%)
Hormonotherapy associated with CDK4/6i	
Anastrozol	1 (1.1%)
Exemestane	3 (3.2%)
Fulvestrant	19 (20.0%)
Letrozol	72 (75.8%)
Menopausal status at initiation of CDK4/6i	
Premenopause	17 (17.9%)
Menopause	78 (82.1%)
Ki67 Percentage	
<20%	43 (45.3%)
>20%	52 (54.7%)
Associated comorbidities	
No	16 (16.8%)
Yes	79 (83.2%)
Family history of cancer	
No	70 (73.7%)
Yes	25 (26.3%)
Death/survival at time of study	
Survival	58 (61.1%)
Death	37 (38.9%)

**Table 2:** Analysis of overall health status, functioning, physical and emotional symptoms, sleep quality, and stress/anxiety levels.

	Median	Minimum	Maximum	Interquartile Range (IQR)	Interpretation
Global health status/QoL	62.7193	16.67	100.0	41.665	Moderate-to-good QoL with significant variability
Physical functioning	52.6	0.00	100.0	50	Moderate physical limitations, with some patients experiencing severe impairments
Role functioning	47.3	0.00	100.0	50	Low functioning in social/occupational roles, highlighting significant difficulties in fulfilling social and occasional responsibilities
Emotional functioning	54.8	0.00	100.0	50	Mediocre emotional well-being with a wide individual variation
Cognitive functioning	64.9	0.00	100.0	50	Relatively better cognitive abilities compared to other domains
Social functioning	50.2	0.00	100.0	50	Low social engagement and potential social isolation for many patients
Fatigue	56.5	0.00	100.0	50	Significant negative impact
Nausea and vomiting	10.0	0.00	66.6	33.3	A rare symptom, but with variations
Pain	24.1	0.00	100.0	50	Experienced at a moderate level, with some patients reporting intense pain
Dyspnoea	13.1	0.00	100.0	50	Moderate to low, but severe for a subset of patients
Insomnia	44.7	0.00	100.0	50	Significant sleep problems affect a large proportion of patients
Appetite loss	25.4	0.00	100.0	50	Moderate, but severe in some patients
Constipation	12.7	0.00	100.0	50	Low in most patients
Diarrhea	14.9	0.00	100.0	50	Similar to constipation, it is a minor problem for most
Financial difficulties	6.5	0.00	66.6	33.3	Low financial strain for most patients, though severe cases exist
Depression	5.4	0	18	9	Some patients scored high
Anxiety	5.0	1	17	8	Moderate anxiety levels with individual variations
Stress	7.9	0	17	8.5	Moderate-to-high stress levels
General fatigue	10.9	7	17	5	A high level of perceived fatigue
Physical fatigue	12.6	9	16	3.5	Consistently reported among most patients
Low activity	13.1	8	16	4	Widespread low physical activity
Low motivation	14.0	10	18	4	Patients report very low motivation, indicating severe emotional and psychological impact
Mental fatigue	11.8	8	17	4.5	Lower than physical fatigue but still significant
Sleep quality score	8.2	3	16	6.5	Indicated a moderate-to-low level of sleep quality; some patients experienced major problems

**Table 3:** Quality of life analysis correlated with CDK4/6i.

Descriptives						
		*n*	Median	Interquartile Range (IQR)	*p*-Value	Interpretation
Global health status/QoL	ribociclib	24	63.9	26.8	0.862	similar scores between groups
	palbociclib	25	61.0			
	abemaciclib	27	63.3			
	Total	76	62.7			
Physical functioning	ribociclib	24	56.4	35.1	0.465	small differences between groups
	palbociclib	25	49.6			
	abemaciclib	27	52.1			
	Total	76	52.6			
Role functioning	ribociclib	24	52.1	40.9	0.612	slightly higher values in the ribociclib group
	palbociclib	25	44.7			
	abemaciclib	27	45.7			
	Total	76	47.4			
Emotional functioning	ribociclib	24	60.1	31.7	0.424	the highest score in the ribociclib group
	palbociclib	25	54.0			
	abemaciclib	27	50.9			
	Total	76	54.8			
Cognitive functioning	ribociclib	24	63.9	37.5	0.907	similar scores between groups
	palbociclib	25	67.3			
	abemaciclib	27	63.6			
	Total	76	64.9			
Social functioning	ribociclib	24	56.3	51.3	0.434	the highest score in the ribociclib group
	palbociclib	25	51.3			
	abemaciclib	27	43.8			
	Total	76	50.2			
Fatigue	ribociclib	24	48.6	35.6	0.189	the lowest fatigue in the ribociclib group
	palbociclib	25	62.7			
	abemaciclib	27	58.0			
	Total	76	56.6			
Nausea and vomiting	ribociclib	24	9.7	24.9	0.500	similar scores between groups
	palbociclib	25	12.0			
	abemaciclib	27	8.6			
	Total	76	10.1			
Pain	ribociclib	24	29.9	39.9	0.389	the highest pain score in the ribociclib group
	palbociclib	25	22.0			
	abemaciclib	27	21.0			
	Total	76	24.1			
Dyspnoea	ribociclib	24	6.9	37.5	0.301	the lowest score in the ribociclib group
	palbociclib	25	14.7			
	abemaciclib	27	17.3			
	Total	76	13.2			
Insomnia	ribociclib	24	30.6	41.3	**0.012**	the lowest score in the ribociclib group; differences are statistically significant, with ribociclib being associated with a lower impact on insomnia
	palbociclib	25	52.0			
	abemaciclib	27	50.6			
	Total	76	44.7			
Appetite loss	ribociclib	24	20.8	40.1	0.504	no significant differences
	palbociclib	25	29.3			
	abemaciclib	27	25.9			
	Total	76	25.4			
Constipation	ribociclib	24	12.5	32.8	0.792	no significant differences
	palbociclib	25	9.3			
	abemaciclib	27	16.0			
	Total	76	12.7			
Diarrhea	ribociclib	24	6.9	34.8	**0.022**	the highest score in the abemaciclib group; differences are statistically significant, with abemaciclib being associated with more frequent diarrhea
	palbociclib	25	10.7			
	abemaciclib	27	25.9			
	Total	76	14.9			
Financial difficulties	ribociclib	24	1.4	23.2	0.144	the lowest score in the ribociclib group
	palbociclib	25	10.7			
	abemaciclib	27	7.4			
	Total	76	6.6			
Depression	ribociclib	19	5.1	5.1	0.872	no significant differences
	palbociclib	20	5.8			
	abemaciclib	21	5.6			
	Total	60	5.5			
Anxiety	ribociclib	19	4.7	4.7	0.717	no significant differences
	palbociclib	20	5.7			
	abemaciclib	21	4.7			
	Total	60	5.0			
Stress	ribociclib	19	8.7	5.5	0.222	no significant differences
	palbociclib	20	8.6			
	abemaciclib	21	6.6			
	Total	60	7.9			
General fatigue	ribociclib	24	11.0	2.6	0.993	no significant differences
	palbociclib	25	10.8			
	abemaciclib	27	11.2			
	Total	76	11.0			
Physical fatigue	ribociclib	24	12.7	2.0	0.705	no significant differences
	palbociclib	25	12.8			
	abemaciclib	27	12.5			
	Total	76	12.7			
Low activity	ribociclib	24	12.9	2.2	0.541	no significant differences
	palbociclib	25	13.2			
	abemaciclib	27	13.4			
	Total	76	13.2			
Low motivation	ribociclib	24	13.5	2.2	0.253	no significant differences
	palbociclib	25	14.3			
	abemaciclib	27	14.2			
	Total	76	14.0			
Mental fatigue	ribociclib	24	11.7	2.2	0.902	no significant differences
	palbociclib	25	12.1			
	abemaciclib	27	11.9			
	Total	76	11.9			
Sleep quality score	ribociclib	24	7.4	4.2	**0.019**	with lower scores in the ribociclib group; differences are statistically significant, with ribociclib being associated with better sleep quality
	palbociclib	25	9.7			
	abemaciclib	27	7.6			
	Total	76	8.2			

**Table 4:** Quality of life analysis correlated with patient evolution.

Group Statistics						
	Death/Survival at the Moment of the Study	*n*	Mean	Interquartile Range (IQR)	*p*-Value	Interpretation
Global health status/QoL	survival	57	64.0	27.5	0.273	The mean is higher for the group of survivors compared to the deceased
	death	19	58.8	24.9		
Physical functioning	survival	57	51.6	36.1	0.539	Survivors have a lower mean compared to the deceased
	death	19	55.8	31.9		
Role functioning	survival	57	48.0	43.7	0.864	The mean values are similar between groups
	death	19	45.6	31.7		
Emotional functioning	survival	57	54.4	34.4	0.776	The mean is almost similar between survivors and the deceased
	death	19	56.1	22.4		
Cognitive functioning	survival	57	65.5	39.1	0.404	The mean is slightly higher in survivors compared to the deceased
	death	19	63.2	33.2		
Social functioning	survival	57	51.2	54.9	0.599	No significant differences
	death	19	47.4	39.1		
Fatigue	survival	57	53.4	37.2	0.094	The deceased group reports a higher score compared to survivors. This is nearly statistically significant.
	death	19	66.1	27.1		
Nausea and vomiting	survival	57	8.5	23.7	0.088	The deceased group has a higher score compared to survivors. This is nearly significant.
	death	19	14.9	27.9		
Pain	survival	57	24.6	41.7	0.773	No significant differences
	death	19	22.8	35.3		
Dyspnoea	survival	57	14.0	40.7	0.897	No significant differences
	death	19	10.5	26.1		
Insomnia	survival	57	43.9	45.0	0.541	No significant differences
	death	19	47.4	27.2		
Appetite loss	survival	57	22.2	41.0	**0.036**	Deceased patients have a higher score compared to survivors. The difference is statistically significant.
	death	19	35.1	35.1		
Constipation	survival	57	12.3	33.6	0.586	No significant differences
	death	19	14.0	31.1		
Diarrhea	survival	57	13.5	36.9	0.063	The survivors have a higher score compared to the deceased group. This is nearly significant.
	death	19	19.3	27.2		
Financial difficulties	survival	57	6.4	24.7	0.438	No significant differences
	death	19	7.0	18.9		
Depression	survival	54	5.7	5.1	0.185	No significant differences
	death	6	3.7	3.9		
Anxiety	survival	54	5.1	4.8	0.663	No significant differences
	death	6	4.0	2.9		
Stress	survival	54	8.1	5.5	0.249	No significant differences
	death	6	6.2	5.6		
General fatigue	survival	57	11.1	2.8	0.275	No significant differences
	death	19	10.5	1.7		
Physical fatigue	survival	57	12.7	2.1	0.829	No significant differences
	death	19	12.7	1.4		
Low activity	survival	57	13.0	2.2	0.175	No significant differences
	death	19	13.7	1.4		
Low motivation	survival	57	14.0	2.0	0.883	No significant differences
	death	19	14.1	2.2		
Mental fatigue	survival	57	11.6	1.8	**0.003**	The mean is higher in deceased patients compared to survivors. The difference is statistically significant.
	death	19	12.8	2.1		
Sleep quality score	survival	57	8.0	4.4	0.269	The score is slightly higher for deceased patients compared to survivors
	death	19	8.8	3.2		

**Table 5:** Correlation coefficients for survival.

Variable	r_p_b	Interpretation
Global health status/QoL	0.115	A slight positive association; survivors tend to have somewhat higher global health status scores.
Physical functioning	−0.070	A very weak negative association; survivors show marginally lower physical functioning scores.
Role functioning	0.033	Essentially negligible; minimal positive association.
Emotional functioning	−0.032	Essentially negligible; minimal negative association.
Cognitive functioning	0.036	Very weak positive association.
Social functioning	0.043	Very weak positive association.
Fatigue	−0.211	A moderate negative association; survivors report lower fatigue levels.
Nausea and vomiting	−0.151	A slight-to-moderate negative association; survivors experience less nausea/vomiting.
Pain	0.026	Negligible association.
Dyspnoea	0.054	Very weak positive association.
Insomnia	−0.049	Very weak negative association.
Appetite loss	−0.189	A slight-to-moderate negative association; survivors report less appetite loss.
Constipation	−0.031	Negligible negative association.
Diarrhea	−0.098	A weak negative association.
Financial difficulties	−0.015	Essentially negligible.
Depression	0.162	A moderate positive association; survivors report higher depression scores.
Anxiety	0.098	A weak positive association; survivors have slightly higher anxiety.
Stress	0.142	A moderate positive association; survivors report higher stress levels.
General fatigue	0.137	A moderate positive association; survivors tend to report higher general fatigue.
Physical fatigue	−0.026	Essentially negligible.
Low activity	−0.180	A moderate negative association; survivors tend to report lower “low activity” (i.e., better activity levels).
Low motivation	−0.008	Virtually no association.
Mental fatigue	−0.363	A moderate-to-strong negative association; survivors report substantially lower mental fatigue.
Sleep quality score	−0.108	A weak negative association; survivors tend to have slightly better sleep quality.

**Table 6:** Quality of life analysis correlated with comorbidities.

Group Statistics						
	Associated Comorbidities	*n*	Mean	Interquartile Range (IQR)	*p*-Value	Interpretation
Global health status/QoL	No	10	70.0	16.9	0.257	The mean is higher for those without comorbidities
	Yes	66	61.6	27.9		
Physical functioning	No	10	61.3	16.9	0.392	Those without comorbidities have higher scores
	Yes	66	51.3	36.7		
Role functioning	No	10	58.3	40.1	0.170	Those without comorbidities have higher scores
	Yes	66	45.7	40.9		
Emotional functioning	No	10	56.7	27.9	0.774	No significant differences
	Yes	66	54.5	32.4		
Cognitive functioning	No	10	70.0	23.2	0.753	No significant differences
	Yes	66	64.1	39.3		
Social functioning	No	10	46.7	50.6	0.766	No significant differences
	Yes	66	50.8	51.7		
Fatigue	No	10	44.4	32.4	0.091	No significant differences
	Yes	66	58.4	35.6		
Nausea and vomiting	No	10	1.7	7.2	0.114	No significant differences
	Yes	66	11.4	26.2		
Pain	No	10	11.7	18.5	0.247	No significant differences
	Yes	66	26.0	41.9		
Dyspnoea	No	10	3.3	14	0.251	No significant differences
	Yes	66	14.6	39.6		
Insomnia	No	10	23.3	21.7	**0.014**	Scores are significantly lower for those without comorbidities. The difference is statistically significant.
	Yes	66	48.0	41.9		
Appetite loss	No	10	16.7	31.9	0.355	No significant differences
	Yes	66	26.8	41.2		
Constipation	No	10	0.0	0.0	**0.046**	The difference is statistically significant
	Yes	66	14.6	34.6		
Diarrhea	No	10	16.7	31.9	0.608	No significant differences
	Yes	66	14.6	35.4		
Financial difficulties	No	10	13.3	37.9	0.481	No significant differences
	Yes	66	5.6	20.3		
Depression	No	7	3.4	4.5	0.075	Those without comorbidities have lower scores compared to those with comorbidities. This is nearly significant.
	Yes	53	5.8	5.1		
Anxiety	No	7	4.4	4.3	0.684	Scores are similar between the two groups
	Yes	53	5.1	4.7		
Stress	No	7	5.7	5.0	0.121	Those without comorbidities have lower scores compared to those with comorbidities. This is nearly significant.
	Yes	53	8.2	5.5		
General fatigue	No	10	10.5	2.3	0.317	Those without comorbidities report less fatigue than those with comorbidities. The difference is nearly significant.
	Yes	66	11.1	2.7		
Physical fatigue	No	10	12.1	2.4	0.150	No significant differences
	Yes	66	12.8	1.9		
Low activity	No	10	12.5	2.3	0.188	No significant differences
	Yes	66	13.3	2.2		
Low motivation	No	10	13.7	1.8	0.557	No significant differences
	Yes	66	14.1	2.2		
Mental fatigue	No	10	12.3	2.2	0.412	No significant differences
	Yes	66	11.8	2.0		
Sleep quality score	No	10	7.5	3.5	0.426	No significant differences
	Yes	66	8.3	4.2		

**Table 7:** Impact of COVID-19 infection on time on treatment (ToT), progression-free survival (PFS), and overall survival (OS) in patients receiving CDK4/6 inhibitors.

COVID Infection		*n*	Mean	Degrees of Freedom (df)
ToT	No	71	13.44	
	Yes	24	26.00	93
	Total	95	16.61	
PFS	No	18	18.67	
	Yes	9	18.89	25
	Total	27	18.74	
OS	No	26	11.85	
	Yes	11	18.82	35
	Total	37	13.92	

**Table 8:** Statistical comparison of time on treatment (ToT), progression-free survival (PFS), and overall survival (OS) between patients with and without COVID-19 infection.

Test Statistics ^a^			
	Mann–Whitney U	*p*-Value (2-Tailed)	Degrees of Freedom (df)
ToT	482,500	0.002	93
PFS	78,000	0.877	25
OS	106,000	0.218	35

^a^ Grouping variable: COVID infection.

## Data Availability

Data are available upon request from the first author, due to privacy and ethical restrictions.

## Supplementary Materials

The following supporting information can be downloaded at: https://www.mdpi.com/article/10.3390/cancers17050818/s1, Table S1: The Kruskal–Wallis H test statistics which compares medians between groups for the quality of life analysis correlated with CDK4/6i. Table S2: The Kruskal–Wallis H test statistics which compares medians between groups for the quality of life analysis correlated with patient evolution. Table S3: The Kruskal–Wallis H test statistics which compares medians between groups for the quality of life analysis correlated with comorbidities. All the four questionnaires can also be found in this section, both in English and in Romanian.
