# Supplementary material for: Real-World Quality-of-Life Data in Metastatic Breast Cancer Patients Treated with CDK4/6 Inhibitors Using Four Assessment Tools
Source: Cancers (Basel). 2025 Feb 26;17(5):818. doi: 10.3390/cancers17050818 (PMC11899285; doi:10.3390/cancers17050818)
Supplement: Supplementary file 1 [file cancers-17-00818-s001.zip › cancers-3483021-supplementary/DASS-21 romanian.pdf]

DASS-21

<u>Instrucţiuni:</u>

Vă rugăm să citiţi fiecare din afirmaţiile de mai jos şi să încercuiţi în dreptul fiecăreia una din cifrele 0, 1, 2 sau 3, pe aceea care consideraţi că exprimă cel mai bine măsura în care afirmaţia respectivă este adevărată în cazul Dvs., în ceea ce priveşte *ultima săptămână*. Nu există răspunsuri corecte sau greşite. Nu petreceţi prea mult timp la niciuna dintre afirmaţii.

Scala de apreciere este după cum urmează:

0 – nu s-a întâmplat deloc, în cazul meu
1- s-a întâmplat într-o anumită măsură, sau doar uneori
2- s-a întâmplat într-o măsură considerabilă, sau într-o destul de mare parte din timp
3- s-a întâmplat foarte mult, sau în cea mai mare parte a timpului

1. Mi-a fost greu să mă calmez 0 1 2 3
2. Am avut senzaţia de uscăciune a gurii 0 1 2 3
3. Parcă n-am putut trăi sentimente pozitive deloc 0 1 2 3
4. Am avut dificultăţi de respiraţie. (ex.: respiraţie excesiv de rapidă, lipsă de aer, în absenţa efortului fizic) 0 1 2 3
5. Mi-a fost greu să mă adun pentru a lua iniţiativa de a face ceva..0 1 2 3
6. Am avut tendinţa de a reacţiona exagerat la diferite situaţii..0 1 2 3
7. Am experienţiat tremurături (de ex., în mâini) 0 1 2 3
8. Am simţit că folosesc foarte multă energie nervoasă 0 1 2 3
9. Am fost îngrijorat(ă) de situaţiile în care m-aş fi putut panica şi face de râs............0 1 2 3
10. Am simţit că nu am nimic ce să aştept..0 1 2 3
11. M-am trezit că devin agitat(ă) 0 1 2 3
12. Mi-a fost greu să mă relaxez 0 1 2 3
13. M-am simţit deprimat(ă) şi trist(ă). 0 1 2 3
14. Am fost intolerant(ă) cu orice lucru care mă împiedica să-mi văd de treabă (adică să continui ceea ce tocmai făceam) 0 1 2 3
15. Am simţit că am fost aproape de a mă panica..0 1 2 3
16. Nu m-am putut entuziasma referitor la nimic 0 1 2 3
17. Am simţit că nu valorez mult, ca persoană..0 1 2 3
18. Am simţit că sunt cam sensibil(ă) 0 1 2 3
19. Am fost conştient(ă) de activitatea inimii mele (observând, de ex., o rată crescută a pulsului, sau lipsa unei bătăi), în absenţa unui efort fizic..0 1 2 3
20. M-am simţit speriat(ă) fără niciun motiv serios 0 1 2 3
21. Am simţit că viaţa e lipsită de sens..0 1 2 3
