# Supplementary material for: Real-World Quality-of-Life Data in Metastatic Breast Cancer Patients Treated with CDK4/6 Inhibitors Using Four Assessment Tools
Source: Cancers (Basel). 2025 Feb 26;17(5):818. doi: 10.3390/cancers17050818 (PMC11899285; doi:10.3390/cancers17050818)
Supplement: Supplementary file 1 [file cancers-17-00818-s001.zip › cancers-3483021-supplementary/MFI english.pdf]

MFI® MULTIDIMENSIONAL FATIGUE INVENTORY

® E. Smets, B.Garssen, B. Bonke.

Instructions:

By means of the following statements we would like to get an idea of how you have been feeling **lately**. There is, for example, the statement:

"I FEEL RELAXED"

If you think that this is **entirely true**, that indeed you have been feeling relaxed lately, please, place an **X** in the extreme left box; like this:

yes, that is true ☒1 ❑2 ❑3 ❑4 ❑5 no, that is not true

The more you **disagree** with the statement, the more you can place an **X** in the direction of "no, that is not true". Please do not miss out a statement and place only one **X** in a box for each statement.

1	I feel fit.	yes, that is true	❑1	❑2	❑3	❑4	❑5	no, that is not true
2	Physically, I feel only able to do a little.	yes, that is true	❑1	❑2	❑3	❑4	❑5	no, that is not true
3	I feel very active.	yes, that is true	❑1	❑2	❑3	❑4	❑5	no, that is not true
4	I feel like doing all sorts of nice things.	yes, that is true	❑1	❑2	❑3	❑4	❑5	no, that is not true
5	I feel tired.	yes, that is true	❑1	❑2	❑3	❑4	❑5	no, that is not true
6	I think I do a lot in a day.	yes, that is true	❑1	❑2	❑3	❑4	❑5	no, that is not true
7	When I am doing something, I can keep my thoughts on it.	yes, that is true	❑1	❑2	❑3	❑4	❑5	no, that is not true
8	Physically I can take on a lot.	yes, that is true	❑1	❑2	❑3	❑4	❑5	no, that is not true
9	I dread having to do things.	yes, that is true	❑1	❑2	❑3	❑4	❑5	no, that is not true
10	I think I do very little in a day.	yes, that is true	❑1	❑2	❑3	❑4	❑5	no, that is not true
11	I can concentrate well.	yes, that is true	❑1	❑2	❑3	❑4	❑5	no, that is not true
12	I am rested.	yes, that is true	❑1	❑2	❑3	❑4	❑5	no, that is not true
13	It takes a lot of effort to concentrate on things.	yes, that is true	❑1	❑2	❑3	❑4	❑5	no, that is not true
14	Physically I feel I am in a bad condition.	yes, that is true	❑1	❑2	❑3	❑4	❑5	no, that is not true
15	I have a lot of plans.	yes, that is true	❑1	❑2	❑3	❑4	❑5	no, that is not true
16	I tire easily.	yes, that is true	❑1	❑2	❑3	❑4	❑5	no, that is not true
17	I get little done.	yes, that is true	❑1	❑2	❑3	❑4	❑5	no, that is not true
18	I don't feel like doing anything.	yes, that is true	❑1	❑2	❑3	❑4	❑5	no, that is not true
19	My thoughts easily wander.	yes, that is true	❑1	❑2	❑3	❑4	❑5	no, that is not true
20	Physically I feel I am in an excellent condition.	yes, that is true	❑1	❑2	❑3	❑4	❑5	no, that is not true
