# Supplementary material for: Real-World Quality-of-Life Data in Metastatic Breast Cancer Patients Treated with CDK4/6 Inhibitors Using Four Assessment Tools
Source: Cancers (Basel). 2025 Feb 26;17(5):818. doi: 10.3390/cancers17050818 (PMC11899285; doi:10.3390/cancers17050818)
Supplement: Supplementary file 1 [file cancers-17-00818-s001.zip › cancers-3483021-supplementary/Pittsburgh Sleep Quality Index english.pdf]

Subject's Initials________________ID#__________________________Date_________________Time_____AM PM

PITTSBURGH SLEEP QUALITY INDEX

INSTRUCTIONS:
The following questions relate to your usual sleep habits during the past month only. Your answers should indicate the most accurate reply for the majority of days and nights in the past month. Please answer all questions.

1. During the past month, what time have you usually gone to bed at night?

   BED TIME ___________

2. During the past month, how long (in minutes) has it usually taken you to fall asleep each night?

   NUMBER OF MINUTES ___________

3. During the past month, what time have you usually gotten up in the morning?

   GETTING UP TIME ___________

4. During the past month, how many hours of actual sleep did you get at night? (This may be different than the number of hours you spent in bed.)

   HOURS OF SLEEP PER NIGHT ___________

For each of the remaining questions, check the one best response. Please answer all questions.

5. During the past month, how often have you had trouble sleeping because you . . .

a) Cannot get to sleep within 30 minutes

Not during the past month_____ Less than once a week_____ Once or twice a week_____ Three or more times a week_____

b) Wake up in the middle of the night or early morning

Not during the past month_____ Less than once a week_____ Once or twice a week_____ Three or more times a week_____

c) Have to get up to use the bathroom

Not during the past month_____ Less than once a week_____ Once or twice a week_____ Three or more times a week_____

d) Cannot breathe comfortably

Not during the past month_____	Less than once a week_____	Once or twice a week_____	Three or more times a week_____

e) Cough or snore loudly

Not during the past month_____	Less than once a week_____	Once or twice a week_____	Three or more times a week_____

f) Feel too cold

Not during the past month_____	Less than once a week_____	Once or twice a week_____	Three or more times a week_____

g) Feel too hot

Not during the past month_____	Less than once a week_____	Once or twice a week_____	Three or more times a week_____

h) Had bad dreams

Not during the past month_____	Less than once a week_____	Once or twice a week_____	Three or more times a week_____

i) Have pain

Not during the past month_____	Less than once a week_____	Once or twice a week_____	Three or more times a week_____

j) Other reason(s), please describe____

____

How often during the past month have you had trouble sleeping because of this?

Not during the past month_____	Less than once a week_____	Once or twice a week_____	Three or more times a week_____

6. During the past month, how would you rate your sleep quality overall?

Very good ___________

Fairly good ___________

Fairly bad ___________

Very bad ___________

7. During the past month, how often have you taken medicine to help you sleep (prescribed or "over the counter")?

   Not during the past month_____ Less than once a week_____ Once or twice a week_____ Three or more times a week_____

8. During the past month, how often have you had trouble staying awake while driving, eating meals, or engaging in social activity?

   Not during the past month_____ Less than once a week_____ Once or twice a week_____ Three or more times a week_____

9. During the past month, how much of a problem has it been for you to keep up enough enthusiasm to get things done?

   No problem at all __________

   Only a very slight problem __________

   Somewhat of a problem __________

   A very big problem __________

10. Do you have a bed partner or room mate?

   No bed partner or room mate __________

   Partner/room mate in other room __________

   Partner in same room, but not same bed __________

   Partner in same bed __________

If you have a room mate or bed partner, ask him/her how often in the past month you have had . . .

a) Loud snoring

   Not during the past month_____ Less than once a week_____ Once or twice a week_____ Three or more times a week_____

b) Long pauses between breaths while asleep

   Not during the past month_____ Less than once a week_____ Once or twice a week_____ Three or more times a week_____

c) Legs twitching or jerking while you sleep

   Not during the past month_____ Less than once a week_____ Once or twice a week_____ Three or more times a week_____

d) Episodes of disorientation or confusion during sleep

   Not during the past month_____ Less than once a week_____ Once or twice a week_____ Three or more times a week_____

e) Other restlessness while you sleep; please describe____

   ____

   Not during the past month_____ Less than once a week_____ Once or twice a week_____ Three or more times a week_____

This form may only be used for non-commercial education and research purposes. If you would like to use this instrument for commercial purposes or for commercially sponsored research, please contact the Office of Technology Management at the University of Pittsburgh at 412-648-2206 for licensing information.
