# Supplementary material for: Real-World Quality-of-Life Data in Metastatic Breast Cancer Patients Treated with CDK4/6 Inhibitors Using Four Assessment Tools
Source: Cancers (Basel). 2025 Feb 26;17(5):818. doi: 10.3390/cancers17050818 (PMC11899285; doi:10.3390/cancers17050818)
Supplement: Supplementary file 1 [file cancers-17-00818-s001.zip › cancers-3483021-supplementary/Pittsburgh Sleep Quality Index romanian.pdf]

Indicele calității somnului Pittsburgh (PSQI)

Instrucțiuni

Întrebările ce urmează se referă la rutina dumneavoastră legată de somn. Vă rugăm să vă referiți doar la ultima lună calendaristică. Răspunsurile dumneavoastră ar trebui să reflecte cel mai potrivit răspuns atunci când vă gândiți la majoritatea nopților. Vă rugăm să răspundeți la toate întrebările.

1. În ultima lună, la ce ora v-ați culcat? ....
2. În ultima lună cât de mult v-a luat să adormiți (în minute)?.............
3. În ultima lună la ce oră v-ați trezit dimineața?..........
4. În ultima lună câte ore ați dormit pe noapte?.............

Pentru următoarele întrebări vă rugăm să selecționați cea mai potrivită afirmație. Vă rugăm să răspundeți la toate întrebările.

5. During the past month, how often have you had trouble sleeping because you…	Deloc in ultima luna<br>1	Mai putin de odata pe sapt.<br>2	O data sau de doua ori pe sapt.<br>3	De 3 sau mai multe ori pe sapt.<br>4
A. Nu ați reușit să adormiți mai repede de 30 de minute				
B. V-ați trezit în timpul somnului sau v-ați trezit foarte devreme				
C. V-ați trezit pentru că trebuia să mergeți la toaletă				
D. Nu ați putut respira confortabil				
E. Tușiți sau sforăiți				
F. Vă este prea cald				
G. Vă este prea frig				
H. Aveți coșmaruri				
I. Aveți durere				
J. Alte motive (descrieți)				
6. În ultima lună cât de des a fost necesar să luați medicamente pentru a putea adormi?				
7. În ultima lună cât de des ați avut probleme în a sta treaz în timp ce conduceți, luați masa, sau erați angajat în alte activități?				
	Deloc	Foarte puțin	Destul	Foarte
8. În ultima lună cât de greu v-a fost să vă păstrați entuziasmul pentru lucrurile de zi cu zi?				
	Foarte bun	Bun	Destul de rău	Rău
9. În ultima lună cum ați notat calitatea somnului per total?				
10. Împărțiți cu cineva camera în timpul nopții?				
