# Supplementary material for: Real-World Quality-of-Life Data in Metastatic Breast Cancer Patients Treated with CDK4/6 Inhibitors Using Four Assessment Tools
Source: Cancers (Basel). 2025 Feb 26;17(5):818. doi: 10.3390/cancers17050818 (PMC11899285; doi:10.3390/cancers17050818)
Supplement: Supplementary file 1 [file cancers-17-00818-s001.zip › cancers-3483021-supplementary/QLQ-C30 Romanian.pdf]

EORTC QLQ-C30 (version 3)

Ne interesează anumite aspecte despre dvs. şi sănătatea dvs. Vă rugăm să răspundeţi personal la toate întrebările, încercuind numărul care vi se potriveşte cel mai bine. Nu există răspunsuri de tipul „corect" sau „greşit". Informaţiile pe care le furnizaţi vor rămâne strict confidenţiale.

Vă rugăm să completaţi cu iniţialele numelui dvs.: □□□□
Data naşterii dvs. (ziua, luna, anul): □□□□□□□□
Data de astăzi (ziua, luna, anul): 31 □□□□□□□□□□

	Deloc	Puţin	Destul de mult	Foarte mult
1. Depuneţi efort în activităţile mai dificile, cum ar fi căratul unei sacoşe grele de cumpărături, sau al unei valize?	1	2	3	4
2. Depuneţi efort în a face o plimbare <u>lungă</u>?	1	2	3	4
3. Depuneţi efort în a face o <u>scurtă</u> plimbare în afara casei?	1	2	3	4
4. Sunteţi nevoit(ă) să staţi în pat sau pe scaun în timpul zilei?	1	2	3	4
5. Aveţi nevoie să fiţi ajutat(ă) când mâncaţi, vă îmbrăcaţi, vă spălaţi, sau folosiţi toaleta?	1	2	3	4

În cursul săptămânii trecute:

	Deloc	Puţin	Destul de mult	Foarte mult
6. Starea dvs de sănătate v-a limitat capacitatea de a vă realiza activităţile profesionale sau alte activităţi zilnice?	1	2	3	4
7. Starea dvs de sănătate v-a limitat capacitatea de a vă ocupa de pasiunile dvs. sau alte activităţi din timpul liber?	1	2	3	4
8. Aţi avut probleme cu respiraţia?	1	2	3	4
9. Aţi avut dureri?	1	2	3	4
10. Aţi fost nevoit(ă) să vă odihniţi?	1	2	3	4
11. Aţi avut necazuri cu somnul?	1	2	3	4
12. V-aţi simţit slăbit(ă)?	1	2	3	4
13. Aţi fost lipsit(ă) de poftă de mâncare?	1	2	3	4
14. Aţi avut senzaţie de greaţă?	1	2	3	4
15. Aţi vomat?	1	2	3	4
16. Aţi fost constipat(ă)?	1	2	3	4

<u>Vă rugăm continuaţi pe pagina următoare.</u>

**În cursul săptămânii trecute:**	**Deloc**	**Puţin**	**Destul de mult**	**Foarte mult**
17. Aţi avut diaree?	1	2	3	4
18. Aţi fost obosit(ă)?	1	2	3	4
19. A interferat durerea cu activităţile dvs. zilnice?	1	2	3	4
20. Aţi avut dificultăţi în a vă concentra asupra unor lucruri, cum ar fi cititul ziarului sau vizionarea televizorului?	1	2	3	4
21. V-aţi simţit încordat(ă)?	1	2	3	4
22. V-aţi făcut griji?	1	2	3	4
23. V-aţi simtit irascibil(ă)?	1	2	3	4
24. Aţi fost deprimat(ă)?	1	2	3	4
25. Aţi avut dificultăţi în a vă aminti unele lucruri?	1	2	3	4
26. Starea dvs. fizică sau tratamentul medical pe care îl urmaţi a interferat cu viaţa dvs. de <u>familie</u>?	1	2	3	4
27. Starea dvs. fizică sau tratamentul medical pe care îl urmaţi a interferat cu activităţile dvs. <u>sociale</u>?	1	2	3	4
28. Starea dvs. fizică sau tratamentul medical pe care îl urmaţi v-a creat greutăţi financiare?	1	2	3	4

Pentru următoarele întrebări, vă rugăm să încercuiţi numărul de la 1 la 7 care vi se potriveşte cel mai bine

29. Cum aţi evalua starea dvs. generală <u>de sănătate</u> în cursul săptămânii trecute?

1 2 3 4 5 6 7

Foarte proastă Excelentă

30. Cum aţi evalua <u>calitatea</u> generală <u>a vieţii</u> dvs. în cursul săptămânii trecute?

1 2 3 4 5 6 7

Foarte proastă Excelentă

© Copyright 1995 EORTC Grupul de studiu al calităţii vieţii. Toate drepturile rezervate. Version 3.
