# Supplementary material for: Real-World Quality-of-Life Data in Metastatic Breast Cancer Patients Treated with CDK4/6 Inhibitors Using Four Assessment Tools
Source: Cancers (Basel). 2025 Feb 26;17(5):818. doi: 10.3390/cancers17050818 (PMC11899285; doi:10.3390/cancers17050818)
Supplement: Supplementary file 1 [file cancers-17-00818-s001.zip › cancers-3483021-supplementary/Test statistics.pdf]

Test statistics

Test Statistics[a,b]

	Kruskal-Wallis H	df	p
Global health status/QoL	,298	2	,862
Physical functioning	1,531	2	,465
Role functioning	,983	2	,612
Emotional functioning	1,717	2	,424
Cognitive functioning	,194	2	,907
Social functioning	1,670	2	,434
Fatigue	3,331	2	,189
Nausea and vomiting	1,386	2	,500
Pain	1,888	2	,389
Dyspnoea	2,403	2	,301
Insomnia	8,865	2	,012
Appetite loss	1,370	2	,504
Constipation	,466	2	,792
Diarrhoea	7,672	2	,022
Financial difficulties	3,876	2	,144
Depression	,274	2	,872
Anxiety	,664	2	,717
Stress	3,011	2	,222
General fatigue	,013	2	,993
Physical fatigue	,698	2	,705
Low activity	1,228	2	,541
Low motivation	2,747	2	,253
Mental fatigue	,206	2	,902
TOTAL score – sleep quality	7,905	2	,019

a. Kruskal Wallis Test

b. Grouping Variable: Type of CDK4/6i administered

Table 3.1: The Kruskal-Wallis H test statistics which compares means between groups for the quality of life analysis correlated with CDK4/6i

Test Statistics[a]

	Mann-Whitney U	p
Global health status/QoL	451,000	,273
Physical functioning	490,500	,539
Role functioning	527,500	,864
Emotional functioning	518,000	,776
Cognitive functioning	473,500	,404
Social functioning	498,500	,599
Fatigue	403,500	,094
Nausea and vomiting	426,000	,088
Pain	518,500	,773
Dyspnoea	533,500	,897
Insomnia	493,500	,541
Appetite loss	380,000	,036
Constipation	506,500	,586
Diarrhoea	413,500	,063
Financial difficulties	502,000	,438
Depression	108,500	,185
Anxiety	144,500	,663
Stress	115,500	,249
General fatigue	452,000	,275
Physical fatigue	524,000	,829
Low activity	432,000	,175
Low motivation	529,500	,883
Mental fatigue	297,500	,003

TOTAL score – sleep quality	450,000	,269

a. Grouping Variable: death/survival at the moment of the study

Table 4.1: The Kruskal-Wallis H test statistics which compares means between groups for the quality of life analysis correlated with patient evolution

Test Statistics[a]

	Mann-Whitney U	p
Global health status/QoL	257,000	,257
Physical functioning	274,500	,392
Role functioning	242,500	,170
Emotional functioning	311,500	,774
Cognitive functioning	310,000	,753
Social functioning	311,000	,766
Fatigue	221,000	,091
Nausea and vomiting	246,500	,114
Pain	258,000	,247
Dyspnoea	274,500	,251
Insomnia	179,500	,014
Appetite loss	274,500	,355
Constipation	230,000	,046
Diarrhoea	302,500	,608
Financial difficulties	302,000	,481
Depression	108,500	,075
Anxiety	168,000	,684
Stress	118,500	,121
General fatigue	266,000	,317
Physical fatigue	239,000	,150
Low activity	247,000	,188
Low motivation	292,500	,557

Mental fatigue	278,000	,412
TOTAL score – sleep quality	278,500	,426

a. Grouping Variable: Associated comorbidities

Table 5.1: The Kruskal-Wallis H test statistics which compares means between groups for the quality of life analysis correlated with comorbidities.
