# Supplementary material for: Real-World Quality-of-Life Data in Metastatic Breast Cancer Patients Treated with CDK4/6 Inhibitors Using Four Assessment Tools
Source: Cancers (Basel). 2025 Feb 26;17(5):818. doi: 10.3390/cancers17050818 (PMC11899285; doi:10.3390/cancers17050818)
Supplement: Supplementary file 1 [file MFI romanian.pdf › cancers-3483021-supplementary/MFI romanian.pdf]

Chestionar de evaluare a diferitelor aspecte ale oboselii (MFIÒ

Instrucțiuni:
Prin intermediul următoarelor afirmații am dori să ne facem împreună o părere despre cum v-ați simțit <u>în ultimul timp</u>. Iată, de exemplu, o astfel de afirmație:

„MĂ SIMT RELAXAT”

În cazul în care considerați că acest lucru este <u>în întregime adevărat</u>, şi anume că într-adevăr în ultimul timp v-ați simțit relaxat, vă rugăm să notați un **x** în căsuța din capătul din stânga.
da, este adevărat x.

1	Mă simt în formă.	**da, este adevărat**	❑	**2**	❑	❑	❑	**nu, nu este adevărat**
2	Din punct de vedere fizic simt că pot face puține lucruri.	**da, este adevărat**	❑	**2**	❑	❑	❑	**nu, nu este adevărat**
3	Mă simt foarte activ.	**da, este adevărat**	❑	**2**	❑	❑	❑	**nu, nu este adevărat**
4	Am chef să fac tot felul de lucruri plăcute.	**da, este adevărat**	❑	❑	❑	❑	❑	**nu, nu este adevărat**
5	Mă simt obosit.	**da, este adevărat**	❑	**2**	❑	❑	❑	**nu, nu este adevărat**
6	Consider că pot face foarte multe lucruri în timpul unei zile.	**da, este adevărat**	❑	❑	❑	❑	❑	**nu, nu este adevărat**
7	Când fac ceva, pot să mă concentrez în totalitate asupra acelui lucru.	**da, este adevărat**	❑	❑	❑	❑	❑	**nu, nu este adevărat**
8	Am putere şi rezistență fizică să fac multe lucruri.	**da, este adevărat**	❑	❑	❑	❑	❑	**nu, nu este adevărat**
9	Mă sperie ideea de a avea lucruri de făcut.	**da, este adevărat**	❑	❑	❑	❑	❑	**nu, nu este adevărat**
10	Consider că fac foarte puține lucruri în timpul unei zile.	**da, este adevărat**	❑	❑	❑	❑	❑	**nu, nu este adevărat**
11	Am o capacitate bună de concentrare.	**da, este adevărat**	❑	❑	❑	❑	**5**	**nu, nu este adevărat**
12	Mă simt odihnit.	**da, este adevărat**	❑	❑	❑	❑	❑	**nu, nu este adevărat**
13	Trebuie să fac efort pentru a mă concentra asupra a ceea ce am de făcut.	**da, este adevărat**	❑	❑	❑	❑	**5**	**nu, nu este adevărat**
14	Simt că sunt într-o perioadă în care condiția mea fizică este proastă.	**da, este adevărat**	❑	❑	**3**	❑	❑	**nu, nu este adevărat**
15	Am o mulțime de planuri.	**da, este adevărat**	❑	❑	❑	❑	**5**	**nu, nu este adevărat**
16	Obosesc cu uşurință.	**da, este adevărat**	❑	❑	❑	❑	❑	**nu, nu este adevărat**
17	Reuşesc să duc la bun sfârşit puține lucruri.	**da, este adevărat**	❑	❑	❑	❑	❑	**nu, nu este adevărat**
18	Nu am chef să fac nimic.	**da, este adevărat**	❑	❑	❑	❑	❑	**nu, nu este adevărat**
19	Îmi pierd uşor firul gândurilor.	**da, este adevărat**	❑	❑	❑	❑	❑	**nu, nu este adevărat**
20	Mă simt într-o condiție fizică excelentă.	**da, este adevărat**	❑	❑	❑	❑	❑	**nu, nu este adevărat**
